# Characteristics of IgG4-related disease complicated with allergic rhinitis or chronic rhinosinusitis: a large cross-sectional cohort study

**DOI:** 10.1038/s41598-022-15398-x

**Published:** 2022-07-14

**Authors:** Qianyu Shi, Xiaoran Ning, Huijuan Li, Xiangbo Ma, Kunkun Wang, Wenjie Bian, Yuxin Zhang, Jiao Xia, Xiaodan Zheng, Yanying Liu, Zhanguo Li

**Affiliations:** 1 Department of Rheumatology and Immunology, Peking University People’s Hospital, 11, Xizhimen South Street, Beijing, 100044 China; 2 Department of Rheumatology and Immunology, People’s Hospital of Hebei Province, Shijiazhuang, China; 3 Department of Rheumatology and Immunology, Handan First Hospital, Handan, China; 4 Department of Rheumatology and Immunology, Tengzhou Central People’s Hospital, Tengzhou, China; 5 Department of Otorhinolaryngology Head and Neck Surgery, Beijing Friendship Hospital, Capital Medical University, Beijing, China; 6 Department of Pathology, Beijing Friendship Hospital, Capital Medical University, Beijing, China; 7 Department of Rheumatology and Immunology, Beijing Friendship Hospital, Capital Medical University, 95, Yong’an Road, Xicheng District, Beijing, 100050 China

**Keywords:** Rheumatic diseases, Immunology

## Abstract

In clinical practice, we found that IgG4-related disease (IgG4-RD) patients complicated with allergic rhinitis (AR)/chronic rhinosinusitis (CRS) seemed to have unique characteristics different from patients with IgG4-RD alone. In this study, demographic, clinical and laboratory characteristics of IgG4-RD patients complicated with AR/CRS were investigated. We retrospectively analyzed 756 IgG4-RD patients who were recruited in four medical centers from 2009 to 2021. We divided 756 IgG4-RD patients into 2 groups: the case group included IgG4-RD patients complicated with AR/CRS, and the control group included IgG4-RD patients without AR/CRS. 411 patients were complicated with AR/CRS among 756 IgG4-RD patients. Multiple organs involvement (≥ 3, *p* < 0.0001, OR 3.585 (95% CI 2.655–4.839)) and other types of allergic disease (*p* < 0.0001, OR 2.007 (95% CI 1.490–2.693)) were more common in the case group. Patients in the case group had a higher level of serum IgG4 (650 mg/dL vs 385 mg/dL, *p* < 0.0001), IgE (347 mg/dL vs 98 mg/dL, *p* < 0.0001) and ESR (14 mm/h vs 12 mm/h, *p* < 0.05). High IgE level (*p* < 0.01, OR 1.003 (95% CI 1.001–1.005)) and other types of allergic disease (*p* < 0.05, OR 3.196 (95% CI 1.146–8.908)) were risk factors for patients in the case group, in which most patients had nasal manifestations before the diagnosis of IgG4-RD. The median time interval from nasal symptoms appearance to IgG4-RD diagnosis was − 120 and − 90 months for patients complicated with AR and CRS, respectively. IgG4-RD patients are often complicated with AR/CRS and have distinct characteristics, which appear to be a subgroup of IgG4-RD. The data suggests a pathogenic association of IgG4-RD and AR/CRS.

## Introduction

IgG4-related disease (IgG4-RD) is an immune-mediated systemic disorder that is typified by sclerosing lesions in multiple organs^1,2^. The characteristic pathological features of IgG4-RD are lymphocyte and plasmacyte infiltration, storiform fibrosis, and obliterative phlebitis^1,3–5^. IgG4-RD was recognized as isolated entities incipiently and was first described in pancreas in a cohort of Japanese patients in 2001^6^. Subsequent studies found that it was a systemic condition and could affect nearly any anatomic site^7,8^.

Allergic rhinitis (AR) is an inflammatory condition that is resulted from immunoglobulin E (IgE)-mediated mucosal inflammation to inhaled allergens, presenting nasal symptoms like sneezing, nasal obstruction, rhinorrhea and nasal itching^9,10^. AR diagnosis is based on clinical manifestations, signs, and skin prick test as well as serum specific IgE detection indicating allergen-specific IgE. Chronic rhinosinusitis (CRS) is the mucosal inflammation of paranasal sinuses^11,12^. The cardinal manifestations of CRS are mucopurulent drainage, nasal obstruction, decreased sense of smell and facial pain-pressure-fullness.

Sinusitis was first recognized as a complication of extra-pancreatic lesions of autoimmune pancreatitis in 2011^13^. Subsequent studies demonstrated that immunohistochemical examination in some IgG4-RD patients with nasal symptoms showed abundant IgG4-positive plasma cell infiltration in the nasal and paranasal sinus mucosa^14,15^. However, studies about the nasal involvement of IgG4-RD had limited samples and mainly focused on CRS^13–17^. In clinical practice, we found that IgG4-RD patients complicated with AR/CRS seemed to have unique characteristics different from patients with IgG4-RD alone. In this study, we retrospectively analyzed the demographic, clinical and laboratory disparities in the 756 IgG4-RD patients. To our knowledge, this is the largest cross-sectional cohort study comparing the two phenotypes of IgG4-RD with and without CRS/AS. Confirming the characteristics of these two groups could refine the classification of IgG4-RD and suggest the pathogenic association of IgG4-RD and AR/CRS.

## Methods

### Patients

We retrospectively analyzed 756 IgG4-RD patients fulfilled 2020 revised comprehensive diagnostic criteria for IgG4-RD^18^, and 479 (63.4%), 96 (12.7%) and 181 (23.9%) cases were diagnosed as definite (fulfilling all clinical, serological and pathological diagnosis criteria), probable (fulfilling only clinical and pathological diagnosis criteria) and possible IgG4-RD (fulfilling only clinical and serological diagnosis criteria) in all enrolled patients, respectively. The exact clinical, serological and pathological diagnosis criteria are^18^: (1) Clinical diagnosis criteria: One or more organs show diffuse or localized swelling or a mass or nodule characteristic of IgG4-RD. In single organ involvement, lymph node swelling is not included; (2) Serological diagnosis criteria: Serum IgG4 levels greater than 135 mg/dl; (3) Pathological diagnosis criteria: Positivity for two of the following three criteria (a. Dense lymphocyte and plasma cell infiltration with fibrosis; b. Ratio of IgG4-positive plasma cells /IgG-positive cells greater than 40% and the number of IgG4-positive plasma cells greater than 10 per high powered field; c. Typical tissue fibrosis, particularly storiform fibrosis, or obliterative phlebitis). All of the patients enrolled in this study were diagnosed as IgG4-RD based on their extra-nasal symptoms and/or extra-nasal biopsy results, since there is no unified definition of IgG4-related nasal disease. All the patients were recruited in 4 medical centers (Peking University People’s Hospital; People’s Hospital of Hebei Province; Handan First Hospital; and Tengzhou Central People’s Hospital) from 2009 to 2021. AR was diagnosed according to the Allergic Rhinitis and its Impact on Asthma (ARIA) 2008 and BSACI guideline 2017^19,20^: AR diagnosis is based on the concordance between a typical history of allergic symptoms and diagnostic tests. (1) Typical symptoms: Including two or more symptoms such as sneezing, watery mucus, nasal congestion, and nasal itching, and the symptoms persist or accumulate for more than 1 h per day. May be accompanied by eye symptoms such as itching and conjunctival congestion. (2) Signs: Nasal mucosa pale, edema, nasal watery secretions. Nasal endoscopy and sinus CT examinations were performed as appropriate. (3) Diagnostic tests: Skin prick test or serum specific IgE detection were done to assist diagnosis. CRS was diagnosed according to the EPOS 2012 criteria^21^: (1) Having two or more symptoms of nose and the paranasal sinuses inflammation, one of which should be either nasal blockage/obstruction/congestion or nasal discharge (anterior/posterior nasal drip), ± facial pain/pressure, ± reduction or loss of smell; (2) Either endoscopic signs (nasal polyps, and/or mucopurulent discharge primarily from middle meatus, and/or edema/mucosal obstruction primarily in middle meatus) or CT/MRI changes (mucosal changes within the ostiomeatal complex and/or sinuses) that support CRS diagnosis. The clinical diagnosis of AR/CRS was made by otolaryngologists. Due to the retrospective nature, we collected patient nasal manifestations through combination of medical records and questionnaires. Patients diagnosed as IgG4-RD were categorized into the case group if they met the diagnostic criteria of AR^19^ and/or CRS^21^. The control group included IgG4-RD without AR/CRS. In the case group, patients with AR were categorized into Group A, patients with CRS were categorized into Group B, and patients with both AR and CRS were categorized into Group C. 46 patients in the case group underwent nasal tissue biopsies. Among them, 28 patients had numerous IgG4-positive plasma cell infiltration, and 4 patients had storiform fibrosis and/or obliterative phlebitis.

Demographic features like age and gender, and clinical characteristics including clinical manifestation, disease duration and organ involvement were recorded. The time interval from disease onset to diagnosis was defined as the time interval from symptoms onset to the diagnosis of IgG4-RD. Other types of allergic disease included atopic dermatitis, asthma etc. This study was approved by Medical Ethics Committees of the four medical centers that were responsible for patient enrollment (Peking University People’s Hospital; People’s Hospital of Hebei Province; Handan First Hospital; and Tengzhou Central People’s Hospital). Data protection authority and medical research ethical committee gave their approval according to national regulations and in accordance with the Declaration of Helsinki. All methods were performed in accordance with the relevant guidelines and regulations. Informed consent was obtained from all patients enrolled in this study.

### Laboratory studies, image features and histological examinations

Laboratory studies including serum IgG4 level, serum IgE level, C-reactive protein (CRP), erythrocyte sedimentation rate (ESR), eosinophilia, C3, C4 and autoantibodies (including RF and ANAs) were retrospectively collected.

All patients received radiological examinations comprising of ultrasonography, Computed Tomography (CT), or Magnetic resonance imaging (MRI); and partial patients underwent 18F-fuorodeoxyglucose PET-CT for systematic examination.

575 patients carried out tissue biopsy and all tissue biopsy samples were fixed in formalin and embedded in paraffin wax. Then all samples were stained with hematoxylin and eosin and immunocytochemistry (by using antibody against CD3, CD20, IgG, IgG4, CD138 and CD38, respectively).

### Statistical analysis

All statistical analyses were performed by GraphPad Prism 8.0 using descriptive methods, with standard summary statistics including mean (S.D.), median, interquartile range (IQR), and proportions as previously reported^22,23^. Student’s *t* test was used for differences for continuous, normally distributed data; Mann–Whitney test was used for differences for continuous, non-normally distributed data. Categorical variables were processed by *X*^2^ or Fisher's exact tests. Logistic regression analysis with enter method was performed to compare the patients in the case group and control group. Factors with *P* < 0.05 and clinical significance in the univariate analysis were included in the multivariate analysis. *P*-value < 0.05 was deemed as statistically significant.

### Ethics approval

This study was approved by Medical Ethics Committees of the four medical centers that were responsible for patient enrollment (Peking University People’s Hospital; People’s Hospital of Hebei Province; Handan First Hospital; and Tengzhou Central People’s Hospital).

### Consent to publish

All authors of this study have their consent for publication.

## Results

### Demographic characteristics

Demographic characteristics of a total of 756 patients diagnosed as IgG4-RD were listed in Table 1. A total of 408 patients (53.97%) were categorized into the case group for complication of AR/CRS. The rest of 348 patients (46.03%) without AR/CRS were categorized into the control group.

**Table 1:** Demographic and clinical characteristics of 756 IgG4-RD patients.

Characteristics	Case group: IgG4-RD^a^ complicated with AR^b^/CRS^c^				Control group: IgG4-RD without AR/CRS
	Total	Group A^d^	Group B^d^	Group C^d^	
Number of cases, n	408	257	116	35	348
Definite diagnosis, n (%)	257 (62.99%)	153 (59.53%)	82 (70.69%)	22 (62.86%)	222 (63.79%)
Probable diagnosis, n (%)	39 (9.56%)	28 (10.89%)	8 (6.90%)	3 (8.57%)	57 (16.38%)
Possible diagnosis, n (%)	112 (27.45%)	76 (29.57%)	26 (22.41%)	10 (28.57%)	69 (19.83%)
Median age at disease onset, years (IQR^e^)	56 (46–62)	55 (46–62)	55 (45–61)	54 (45.5–61)	56 (46–64)
Median age at diagnosis, years (IQR)	57.5 (50–64)	56 (48–65)	57 (50–64)	57 (49.5–63)	58 (48–65)
Median time from onset to diagnosis, months (IQR)	24 (12–60)	12 (0–36)	18 (0–48)	12 (0–48)	12 (6–36)
Gender (Male: Female)	1.34:1	1.22:1	1.56:1	1.69:1	1.46:1
Other types of allergic disease, n (%)	216 (52.55)	115 (44.75)	85 (56.29)	20 (57.14)	43 (12.36)
**Organ involvement, n (%)**					
1 organ involved	77 (18.87)	63 (24.51)	13 (11.21)	1 (2.86)	138 (39.66)
2 organs involved	74 (18.14)	47 (18.29)	23 (19.83)	4 (11.43)	67 (19.25)
3 organs involved	69 (16.91)	34 (13.23)	26 (22.41)	9 (25.71)	56 (16.09)
≥ 4 organs involved	188 (46.08)	113 (43.97)	54 (46.55)	21 (60.00)	87 (25.00)

^a^IgG4-RD, IgG4-related disease; ^b^AR, allergic rhinitis; ^c^CRS, chronic rhinosinusitis; ^d^Group A included IgG4-RD patients complicated with AR, Group B included IgG4-RD patients complicated with CRS and Group C included IgG4-RD patients complicated with AR and CRS; ^e^IQR, interquartile range.

Patients in the case group had longer time interval from onset to diagnosis (24 months vs 12 months, *p* = 0.0089), longer disease duration (24 months vs 12 months, *p* = 0.0096) and longer follow-up period (51.5 months vs 41 months, *p* = 0.0107) compared with patients in the control group (Table 2).

**Table 2:** Differences of demographic, clinical and laboratory characteristics between IgG4-RD patients complicated with AR/CRS (case group) and without AR/CRS (control group).

Characteristics	ALL (n = 756)	Case group (n = 408)^a^	Control group (n = 348)^a^	*P*- value^b^
**Demographics**				
Age at disease onset, years, median (IQR^c^)	56 (46–63)	56 (46–62)	56 (46–64)	0.4380
Age at diagnosis, years, median (IQR)	57.5 (49–65)	57.5 (50–64)	58 (48–65)	0.5078
Time from onset to diagnosis, months, median (IQR)	24 (12–48)	24 (12–60)	12 (6–36)	0.0089*
Follow-up period, months, median (IQR)	48 (26–71.5)	51.5 (33–72)	41 (24–70)	0.0107*
Disease duration, months, median (IQR)	24 (12–48)	24 (12–60)	12 (6–36)	0.0096*
Gender (Male: Female)	1.39:1	1.34:1	1.46:1	0.6838
**Clinical features n (%)**				
Other types of allergic disease	341 (45.11)	216 (52.55)	125(36.23)	< 0.0001*
Number of involved organs ≥ 3	391 (51.7)	269 (65.5)	122(35.4)	< 0.0001*
Lymph node	262 (34.66)	169 (41.12)	93 (26.96)	< 0.0001*
Thyroid gland	46 (6.08)	30 (7.30)	16 (4.64)	0.1273
Lung	174 (23.02)	123 (29.93)	51 (14.78)	< 0.0001*
Kidney	113 (14.95)	64 (15.57)	49 (14.20)	0.5590
Liver	28 (3.70)	17 (4.14)	11 (3.19)	0.4919
Pancreas	217 (28.70)	122 (29.68)	95 (27.54)	0.5156
Biliary system	96 (12.70)	52 (12.65)	44 (12.75)	0.9667
Gallbladder	67 (8.86)	43 (10.46)	24 (6.96)	0.0911
Retroperitoneal fibrosis	124 (16.40)	61 (14.84)	63 (18.26)	0.1020
Mesentery	7 (0.93)	4 (0.97)	3 (0.87)	0.8822
Aorta	17 (2.25)	13 (3.16)	4 (1.16)	0.0642
Prostate	35 (4.63)	20 (4.87)	15 (4.35)	0.7355
Salivary gland	406 (53.70)	260 (63.26)	146 (42.32)	< 0.0001*
Lacrimal gland	300 (39.68)	214 (52.07)	86 (24.93)	< 0.0001*
Parotid gland	212 (28.04)	146 (35.52)	66 (19.13)	< 0.0001*
**Laboratory findings**				
Serum IgG4 (mg/dL), median (IQR)	502 (248–1360)	650 (312–1520)	385 (185–1136)	< 0.0001*
Serum IgG4 (mg/dL)/IgG (mg/dL), median (IQR)	0.28 (0.14–0.49)	0.32 (0.19–0.53)	0.21 (0.12–0.38)	< 0.0001*
Serum IgE (IU/ml), median (IQR)	246 (72–655)	347 (153–958)	98 (39–288)	< 0.0001*
CRP (mg/dl), median (IQR)	1.83 (0.68–6.40)	1.98 (0.74–6.87)	1.63 (0.61–6)	0.5607
ESR (mm/h), median (IQR)	13 (7–36)	14 (7–42)	12 (6–28)	0.0481*
Elevated CRP, n (%)	117 (15.48)	67 (16.30)	50 (14.50)	0.4934
Elevated ESR, n (%)	228 (30.16)	142 (34.55)	86 (24.93)	0.0041*
Eosinophilia, n (%)	92 (12.17)	73 (17.76)	19 (5.51)	< 0.0001*
C3 (g/L) mean (SD)	0.89 (0.74–1.08)	0.84 (0.73–1.04)	0.97 (0.81–1.12)	< 0.0001*
C4 (g/L), median (IQR)	0.19 (0.15–0.26)	0.19 (0.14–0.24)	0.21 (0.16–0.28)	< 0.0001*
ANA (+), n (%)	94 (12.43)	51 (12.41)	43 (12.46)	0.9818
RF (+), n (%)	97 (12.83)	56 (13.63)	41 (11.88)	0.4758
Hypocomplementemia, n (%)	232 (30.69)	160 (38.93)	72 (20.87)	< 0.0001*

^a^Case group included IgG4-RD patients complicated with AR or CRS, and the control group included IgG4-RD patients without AR or CRS; ^b^*means *P*-value < 0.05; ^c^IQR, interquartile range

### Clinical features

In general, the proportion of number of involved organs ≥ 3 in the case group was higher than that in the control group (65.5% vs 35.4%, *p* < 0.0001) as shown in Table 2. Patients in the case group showed more lymph node (41.12 vs 26.96, *p* < 0.0001), lung (29.93 vs 14.78, *p* < 0.0001), salivary gland (63.26 vs 42.32, *p* < 0.0001), lacrimal gland (52.07 vs 24.93, *p* < 0.0001) and parotid gland (35.52 vs 19.13, *p* < 0.0001) involvement than the control group. The most frequently involved organs in the case group were salivary gland (63.26%), lacrimal gland (52.07%) and lymph node (41.12%). In contrast, salivary gland (42.32%), pancreas (27.54%) and lymph node (26.96%) were the most frequently involved organs in the control group.

### Laboratory findings

Patients in the case group had higher level of serum IgG4 (650 vs 385, p < 0.0001), serum IgE (347 vs 98, *p* < 0.0001) and ESR (14 vs 12, *p* = 0.0481) than control group, as shown in Table 2. Eosinophilia (17.76% vs 5.51%, *p* < 0.0001) and hypocomplementemia (38.93% vs 20.87%, *p* < 0.0001) were more common in the case group, too.

### Histological examinations

**Figure 1:**
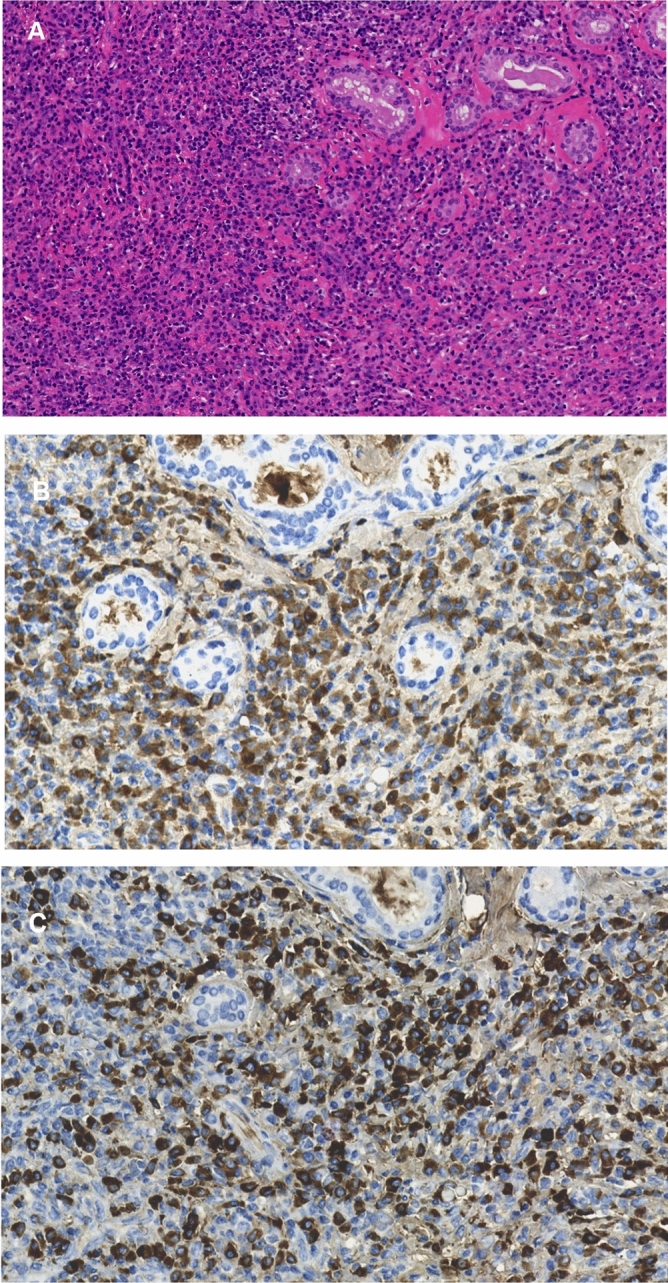
H&E and immunohistochemical staining of nasal mucosa specimens of IgG4-RD patients complicated with CRS. A: H&E staining of nasal mucosa specimens of IgG4-RD patients complicated with CRS showed dense lymphocyte infiltration ((**A**) original magnification 200×). B&C: IgG ((**B**) original magnification 400×) and IgG4 ((**C**) original magnification 400×) immunohistochemical staining of nasal mucosa specimens of IgG4-RD patients complicated with CRS.

46 patients in the case group underwent nasal tissue biopsies. Among them, 28 patients had dense IgG4-positive plasma cells (> 10 per high powered field) infiltration as shown in Fig. 1 and the ratio of IgG4-positive plasma cells /IgG-positive cells is greater than 40%. The other 4 patients had storiform fibrosis and/or obliterative phlebitis that in favor of the diagnosis of IgG4-RD.

### Clinical nasal manifestations of patients in the case group

**Figure 2:**
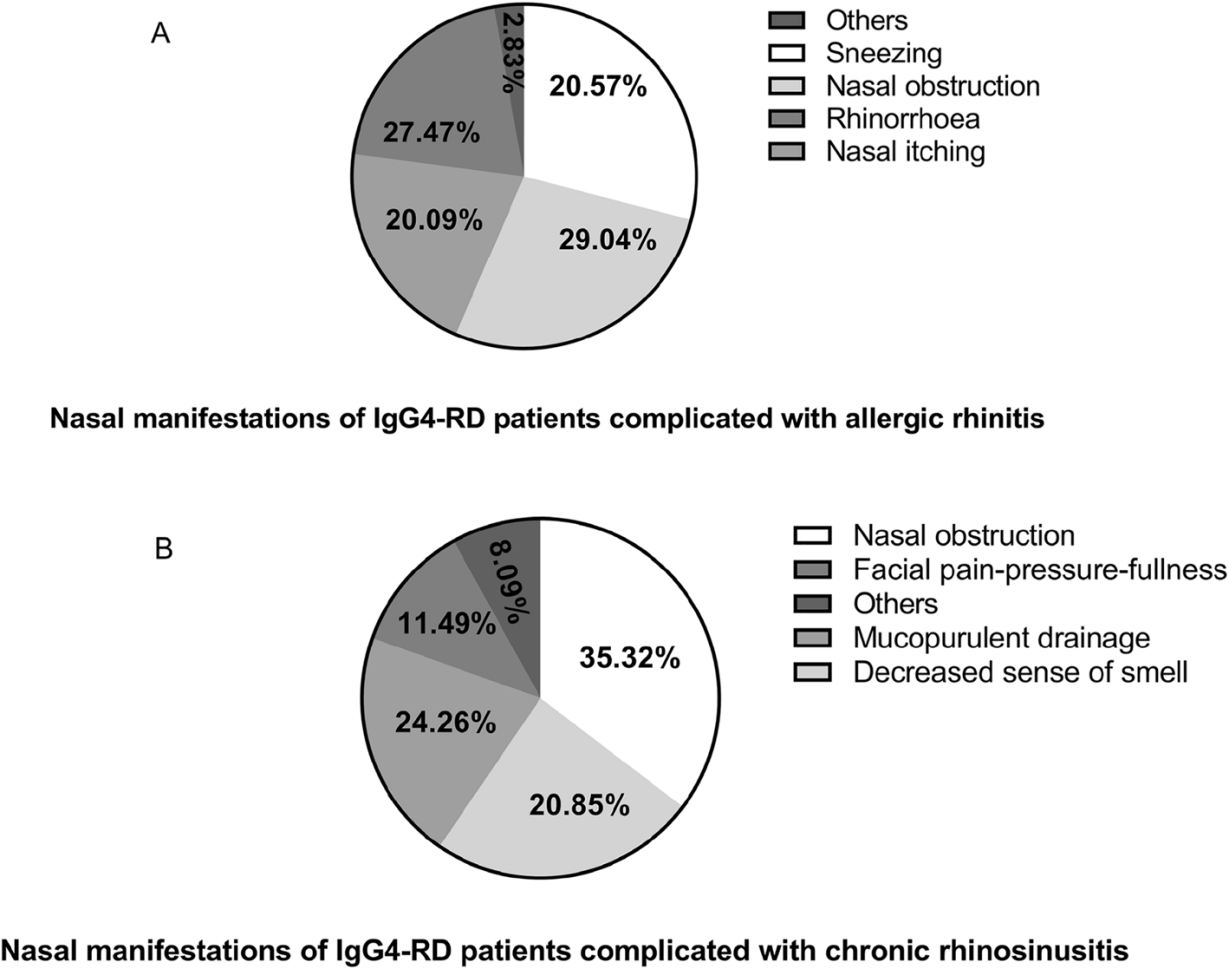
Nasal manifestation of IgG4-RD patients complicated with AR (Group A) and CRS (Group B). (**A**) Patients complicated with AR showed sneezing, nasal obstruction, nasal itching and rhinorrhea most commonly. (**B**) Patients complicated with CRS showed nasal obstruction, decreased sense of smell, mucopurulent drainage and facial pain-pressure-fullness most commonly. IgG4-RD, IgG4-related disease; AR, allergic rhinitis; CRS, chronic rhinosinusitis.

The main clinical nasal manifestations of patients in Group A were sneezing (29.04%), nasal obstruction (27.47%), nasal itching (20.57%) and rhinorrhea (20.09%) as shown in Fig. 2. The main clinical nasal manifestations of patients in Group B were nasal obstruction (35.32%), decreased sense of smell (24.26%), mucopurulent drainage (20.85%) and facial pain-pressure-fullness (11.49%), which were consistent with previous study^14^.

### Risk factors for the patients in the case group

In univariate analysis, disease duration, other types of allergic disease, number of involved organs ≥ 3, lymph node involvement, salivary gland involvement, thyroid gland involvement, lung involvement, biliary system involvement, gallbladder involvement, salivary gland involvement, lacrimal gland involvement, parotid gland involvement, higher level of serum IgG4, higher level of serum IgE, higher level of ESR, eosinophilia and hypocomplementemia were associated with higher rate of IgG4-RD complicated with AR/CRS (Table 3).

**Table 3:** Univariate analysis of logistic regression of risk factors for IgG4-RD patients complicated with AR or CRS (Case group).

Characteristics	Univariate analysis		
	*P*-value^a^	OR	95%CI
**Demographics**			
Age at disease onset, median (IQR^b^)	0.438	0.996	0.985, 1.006
Age at diagnosis, median (IQR)	0.507	1.004	0.993, 1.014
Disease duration, months, median (IQR)	0.011*	1.006	1.001, 1.010
Female, n (%)	0.684	0.941	0.704, 1.259
**Clinical features**			
Other types of allergic disease, n (%)	0.001*	5.093	3.389, 7.655
Organ involvement, n (%)			
≥ 3 organs	0.001*	3.827	2.823, 5.189
Lymph node	0.001*	2.856	2.058, 3.962
Thyroid gland	0.040*	2.011	1.032, 3.919
Lung	0.001*	3.071	2.073, 4.548
Kidney	0.214	1.315	0.853, 2.027
Liver	0.328	1.512	0.660, 3.466
Pancreas	0.202	1.237	0.892, 1.713
Biliary system	0.027*	1.672	1.060, 2.636
Gallbladder	0.017*	2.013	1.134, 3.573
Retroperitoneal fibrosis	0.916	0.979	0.657, 1.458
Mesentery	0.548	1.686	0.307, 9.258
Aorta	0.076	2.785	0.900, 8.620
Prostate	0.736	1.125	0.567, 2.233
Salivary gland	0.001*	2.347	1.751, 3.146
Lacrimal gland	0.001*	3.272	2.396, 4.467
Parotid gland	0.001*	2.329	1.665, 3.258
**Laboratory examinations**			
Serum IgG4 (mg/dL), median (IQR)	0.001*	1.000	1.000, 1.001
Serum IgG4 (mg/dL)/IgG (mg/dL), median (IQR)	0.001*	8.545	2.804, 26.036
Serum IgE (IU/ml), median (IQR)	0.001*	1.002	1.001, 1.002
CRP (mg/dl), median (IQR)	0.428	1.004	0.994, 1.015
ESR (mm/h), median (IQR)	0.001*	1.024	1.003, 1.045
Eosinophilia, n (%)	0.001*	4.509	2.068, 9.831
ANA (+), n (%)	0.115	0.639	0.366, 1.115
RF (+), n (%)	0.937	1.024	0.573, 1.828
Hypocomplementemia, n (%)	0.018*	1.754	1.100, 2.796

^a^*means *P*-value < 0.05; ^b^IQR, interquartile range.

**Figure 3:**
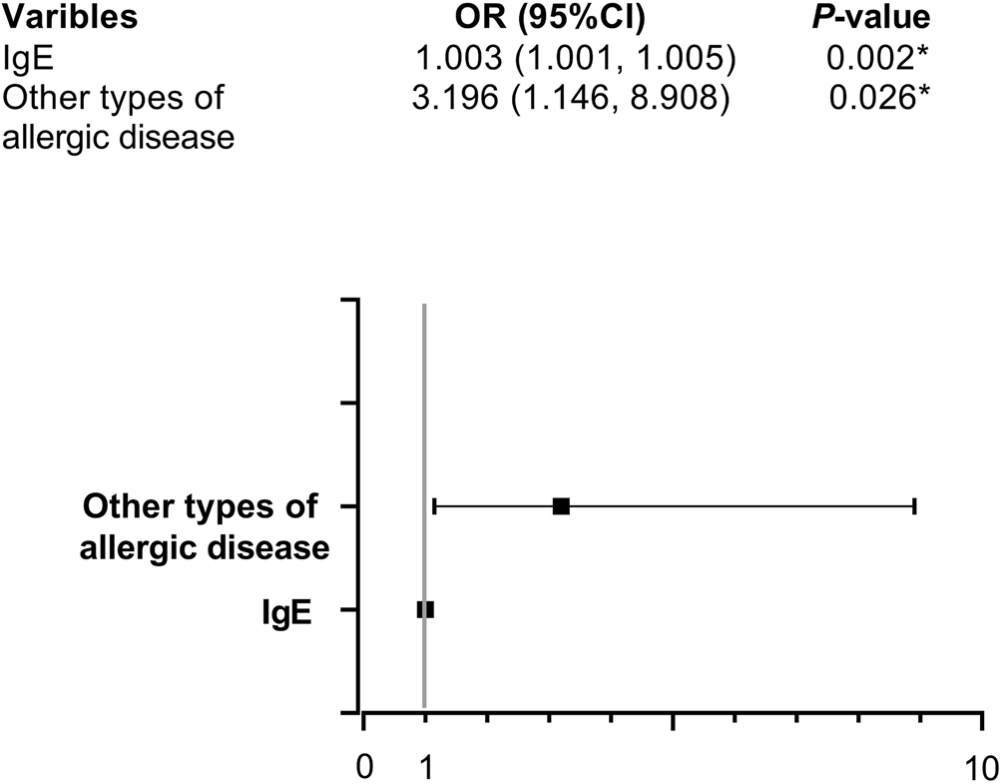
Multivariate analysis of logistic regression of IgG4-RD patients complicated with AR/CRS (Case group). High IgE level and other types of allergic disease were risk factors for patients complicated with AR/CRS. IgG4-RD, IgG4-related disease; AR, allergic rhinitis; CRS, chronic rhinosinusitis; *means *P*-value < 0.05.

In multivariate analysis, only IgE and other types of allergic disease were associated with complication of AR/CRS (Fig. 3).

### Time interval between complication of AR/CRS and disease diagnosis in the case group

The time interval was calculated between the time of complication of AR/CRS and the time of IgG4-RD diagnosis. Positive or negative results meant the complication of AR/CRS occurred after or before IgG4-RD diagnosis, respectively. The time relationship of complication of AR/CRS and IgG4-RD diagnosis was divided into 3 groups including: complication of AR/CRS occurred before IgG4-RD diagnosis, complication of AR/CRS occurred after IgG4-RD diagnosis and complication of AR/CRS occurred simultaneously with IgG4-RD diagnosis (defined as the time interval between complication of AR/CRS and IgG4-RD diagnosis was no more than 12 months).

**Figure 4:**
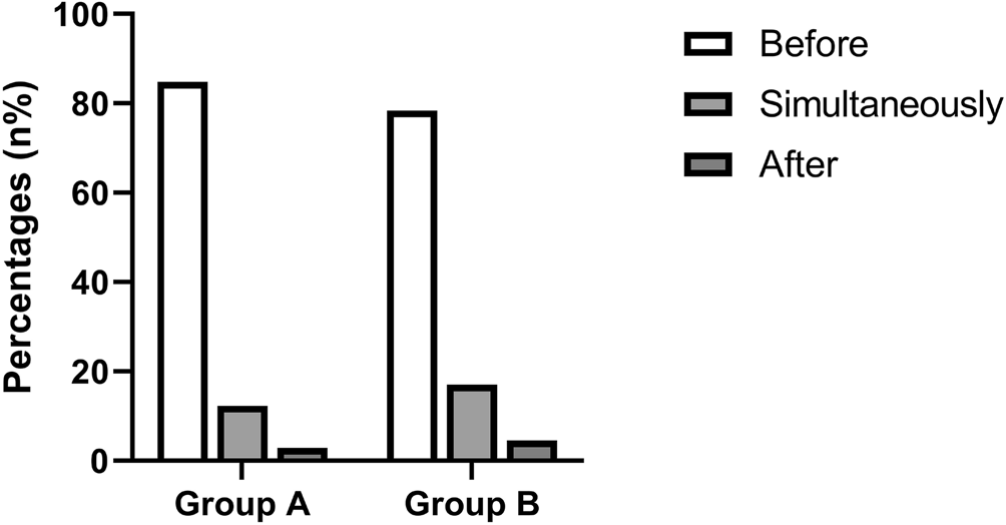
Chronological relationship of complication of AR/CRS and IgG4-RD diagnosis in IgG4-RD patients. In Group A (IgG4-RD complicated with AR), 84.83% patients had nasal symptoms before IgG4-RD diagnosis. In Group B (IgG4-RD complicated with CRS), 78.41% patients had nasal symptoms before IgG4-RD diagnosis. IgG4-RD, IgG4-related disease; AR, allergic rhinitis; CRS, chronic rhinosinusitis.

As shown in Fig. 4, the percentage of each group in Group A was 84.83% (complication of AR/CRS occurred before IgG4-RD diagnosis), 12.32% (complication of AR/CRS occurred simultaneously with IgG4-RD diagnosis) and 2.84% (complication of AR/CRS occurred after IgG4-RD diagnosis), respectively. The percentage of each group in Group B was 78.41% (complication of AR/CRS occurred before IgG4-RD diagnosis), 17.05% (complication of AR/CRS occurred simultaneously with IgG4-RD diagnosis) and 4.55% (complication of AR/CRS occurred after IgG4-RD diagnosis), respectively. The median time interval of each group in Group A was 144 months (IQR: 96–306; complication of AR/CRS occurred before IgG4-RD diagnosis), 9 months (IQR: 2–12; complication of AR/CRS occurred simultaneously with IgG4-RD diagnosis) and 54 months (IQR: 39–69; complication of AR/CRS occurred after disease IgG4-RD diagnosis), respectively. The median time interval of each group in Group B was 120 months (IQR: 60–360; complication of AR/CRS occurred before IgG4-RD diagnosis), 12 months (IQR: 6–12; complication of AR/CRS occurred simultaneously with IgG4-RD diagnosis) and 35.5 months (IQR: 32.25–57; complication of AR/CRS occurred after IgG4-RD diagnosis), respectively.

## Discussion

In the present study, we described the clinical manifestations of IgG4-RD patients complicated with AR/CRS in a large cross-sectional cohort. Moreover, we compared the demographic, clinical and laboratory disparities of 408 IgG4-RD patients complicated with AR/CRS and 348 IgG4-RD patients without AR/CRS. The chronological relationship of complication of AR/CRS and IgG4-RD disease diagnosis was analyzed. To our current knowledge, this is the first and largest cross-sectional cohort study focusing on the complications of both AR and CRS of IgG4-RD, and exploring the differences of IgG4-RD with and without AR/CRS phenotypes.

IgG4-RD is an immune-mediated systemic disorder that is typified by sclerosing lesions in multiple organs. Organs in the head and neck region are commonly involved in IgG4-RD, such as salivary gland, lacrimal gland and parotid gland^22,23^. However, few studies have discussed nasal involvement in IgG4-RD, which is mainly because there is no classification diagnostic criterion for IgG4-related rhinitis or IgG4-related rhinosinusitis. Clinical symptoms of exocrine gland involvement are common in IgG4-RD patients^8^ and secretory gland is a major part of the nasal membrane, which indicates that the nasal membrane may be theoretically involved in IgG4-RD^17^. Moreover, patients with IgG4-RD do have a higher proportion of complication of AR/CRS based on previous study^13^ and our clinical practice experiences. Therefore, this study retrospectively analyzed the demographic, clinical and laboratory characteristics of IgG4-RD patients complicated with AR/CRS, which could be conducive to better understanding of the pathogenesis of IgG4-RD.

The percentage of complication of AR/CRS in the present study was 54.0%, which was higher than previous studies (43.5%^15^, 37.0%^14^ and 32.3%^13^). The different inclusion criteria may explain for the discrepancy. IgG4-RD patients complicated with AR/CRS seemed to have longer time interval from disease onset to diagnosis. Multiple organs involvement (≥ 3 organs) was more common in patients complicated with AR/CRS than patients without AR/CRS, and the distribution of involved organs were also different, suggesting different underlying pathogenesis.

IgG4-RD patients complicated with AR/CRS had higher serum IgG4 level and serum IgE level. Other types of allergic disease and eosinophilia were also more common in these patients because many IgG4-RD patients with AR/CRS had an allergic constitution. IgG4-RD patients complicated with AR/CRS presented more elevated ESR level and hypocomplementemia that indicated high disease activity, which was correlated with more multiple organs involvement.

Higher serum IgE level and other types of allergic disease were risk factors for complication of AR/CRS in IgG4-RD patients. Both factors suggest the existence of allergic disease, which is a known risk factor for AR^24^, and AR was a common concomitant disease of IgG4-RD patients with CRS^13^. We found that most patients in the case group had nasal symptom onset years earlier before IgG4-RD diagnosis, which may be due to the ignorance of underlying relationship of IgG4-RD and AR/CRS.

The relationship of AR/CRS and IgG4-RD is unclear based on previous studies and there is no unified definition of IgG4-related nasal lesions. However, many patients diagnosed as IgG4-RD were complicated with AR/CRS in the present study, which was consistent with previous studies^13–17^.

Our research supports the hypothesis that IgG4-RD patients with AR/CRS may have distinctive characteristics, which is supported by several findings: (1) 54.0% patients (408) in our study had AR/CRS (34.0% for AR, 15.3% for CRS and 4.7% for both), which was higher than the morbidity of general population(20.2% for AR^25^ and 8% for CRS^26^); (2) IgG4-RD patients with AR/CRS showed different demographic, clinical and laboratory characteristics than patients without AR/CRS; (3) IgG4-RD patients with AR/CRS in our study were sensitive to glucocorticoid therapy and their nasal symptoms alleviated or disappeared after treatment, while CRS alone is insensitive to glucocorticoid therapy. Although AR alone is sensitive to glucocorticoid therapy, IgG4-RD patients complicated with AR had outstanding allergic symptoms and showed different laboratory characteristics than patients with IgG4-RD alone. Therefore, we consider AR may be related to IgG4-RD. However, we couldn’t draw a definite conclusion from the data. Our study may draw attention to the nasal involvement of IgG4-RD, which was barely reported before. Future studies should focus more on the pathological changes between IgG4-RD complicated with AR/CRS, IgG4-RD alone, and AR/CRS alone, which may lead to the recognition of a new clinical entity of IgG4-RD.

There are several limitations of this study. First, although most cases underwent general examinations, its retrospective nature made some involved organs neglected. Second, due to the long time-interval of complication of AR/CRS and IgG4-RD diagnosis, some nasal manifestations of IgG4-RD patients with AR/CRS may be neglected because of recall bias.

## Conclusion

In the present study, we compared the demographic, clinical and laboratory differences between IgG4-RD patients with and without AR/CRS. IgG4-RD patients with AR/CRS had longer time interval from onset to diagnosis. Other types of allergic disease and multiple organs involvement were more common in these patients. IgG4-RD patients with AR/CRS showed higher levels of serum IgG4 and IgE, and higher percentages of ESR elevation, eosinophilia and hypocomplementemia. Physicians should pay attention to the medical history of AR/CRS because AR/CRS could occur several years earlier than IgG4-RD diagnosis in the present study. IgG4-RD patients are often complicated with AR/CRS, which may have distinctive characteristics. The data in this study suggests a pathogenic association of IgG4-RD and AR/CRS.

## Data Availability

The datasets generated during and/or analysed during the current study are available from the corresponding author on reasonable request.
